# Exploring the gut microbiota of healthy captive Asian elephants from various locations in Yunnan, China

**DOI:** 10.3389/fmicb.2024.1403930

**Published:** 2024-09-27

**Authors:** Yuhan Wang, Yixuan Wang, Jiuxuan Zhou, Mingwei Bao, Taif Shah, Song Yang, Jing Zheng, Qian Li, Yutong Hou, Binghui Wang, Ruiling Yuan

**Affiliations:** ^1^Yunnan Academy of Forestry and Grassland, Kunming, China; ^2^Southwest Forestry University, Kunming, China; ^3^Xishuangbanna Wild Elephant Valley, Kunming, China; ^4^Kunming University of Science and Technology, Kunming, China; ^5^School of Public Health, Kunming Medical University, Kunming, China

**Keywords:** *Elephas maximus*, captivity, microbiota, rescue of wild, gut

## Abstract

**Introduction:**

The Asian elephant (*Elephas maximus*) is a giant herbivore classified as an endangered wildlife species by the International Union for Conservation of Threatened Species.This study aims to investigate and compare the core gut microbiota of captive Asian elephants from three different locations in Yunnan Province, China, to explore the impact of environmental and husbandry factors on microbial diversity.

**Methods:**

We collected fecal samples from 29 captive Asian elephants from three locations and performed full-length 16S rRNA gene sequencing. Microbial diversity was assessed using alpha diversity (Chao1 and Shannon indexes) and beta diversity (Bray-Curtis and Euclidean distance metrics). Principal coordinate analysis (PCoA) was used to visualize microbial variation among groups.

**Results:**

Alpha diversity analysis showed that the microbial diversity in the Yexianggu group was higher than that in the other groups. Bray-Curtis and Euclidean metrics revealed significant differences among the microbial communities. Bacteroidetes and Firmicutes, which are key cellulose-degrading bacteria, were the dominant phyla in all groups. Synergistaceae was the most abundant family in the Menghai group, while Lachnospiraceae and Pirellulaceae were more abundant in the Yexianggu and Yuantongshan groups, respectively. Genus *p-1008-a5-gut-group* was more abundant in Yexianggu, and Prevotella was predominant in Menghai.

**Discussion:**

These results indicate that habitat and husbandry practices significantly influence the gut microbiota of captive Asian elephants. The identification of bacterial species such as *Lactobacillus fermentum*, *Clostridium neonatale*, *Enterococcus mundtii*, *Klebsiella huaxiensis*, *Corynebacterium nasicanis*, and *Streptococcus equinus* highlights the potential role of specific microbes in maintaining host-microbial interactions. Promoting microbial diversity through improved captive conditions could enhance the health of these endangered animals.

## Introduction

The Asian elephant (*Elephas maximus*) is one of the largest herbivorous animals, mainly found in Yunnan Province, China, and some parts of south and southeast Asia (Li et al., 2023). These animals are classified as endangered by the International Union for Conservation of Threatened Species and are protected as Class I wildlife species in China (Williams et al., 2020). In addition, Asian elephants are listed in Appendix I of the Convention on International Trade in Endangered Species of Wild Fauna and Flora. In China, improving laws and regulations for protecting critically endangered wildlife, especially Asian elephants, has achieved results (Shi et al., 2021; Chen et al., 2023). After years of effort, the Asian elephant population has rebounded, but its survival rate still requires improvement. So far, several captive Asian elephants rescued from the wild in China have weaker survival abilities than wild populations, i.e., captive elephants often experience gastrointestinal disorders, lameness, stiffness, low fertility, and high mortality rates (Morfeld et al., 2016). Furthermore, no unified captive management system exists for raising Asian elephant populations in different regions (Fraser, 2008). Therefore, the population protection of the Asian elephant is related to the captive management system, but we should also pay more attention to health problems such as gut microflora.

Trillions of microbes in the mammalian gastrointestinal tracts play an essential role in maintaining host hemostasis (Davidar et al., 2023) by preventing the overgrowth of pathogenic bacteria and regulating immunity, metabolism, and ecological adaptation (Davidar et al., 2023). Microbiome profiles are specific to each individual and are influenced by factors such as diet, age, and geographical location (Budd et al., 2020). Diets have been reported as one of the key factors influencing microbial diversity in the gut of healthy elephants. Microbial fermentation in the lower digestive tract enables elephants to gain energy from indigestible plant diets by degrading cellulose and hemicellulose into monosaccharides (Struckmann Poulsen et al., 2022). The dominant bacteria taxa, Oscillospiraceae, Proteobacteria, Bacteroidetes, Actinobacteria, Verrucomicrobia, and Fibrobacteres (Budd et al., 2020), have been involved in the fermentation processes. Firmicutes and hemicellulose-degrading bacteria were abundant in wild and captive Asian elephants, indicating a high digestibility of complex food materials in these herbivores (Li et al., 2022). A study explored diverse microbial profiles in the gut of several captive elephants using full-length 16S rRNA gene sequencing. Several uncultured bacteria taxa, including *Bacteroides, Lachnospira*, *Phascolarctobacterium*, and *Rikenella*, were abundant in elephants fed solid food materials. The study reports distinct microbial profiles among adult elephants fed with local palm and *Caryota urens*. Potential beneficial bacteria were revealed in the gut samples (Klinsawat et al., 2023), providing baseline microbial profiles for monitoring elephant health. Moreover, a study of wild African elephants (Kartzinel et al., 2019) showed that seasonal food availability correlates with the diversity of gut microbial composition.

Some studies have shown that the captivity of wild animals may alter gut microbiota composition and abundance, which may lead to an increase in potential pathogens in the gut microbiota and even affect the development and behavior of wild animals (Moustafa et al., 2021; Li et al., 2022). Moreover, observing a specific environment that influences gut microbiota was only a preliminary observation (Kandel et al., 2020). There is relatively little literature on the gut microbiota of Asian elephants. Therefore, studying the gut microbiota of Asia elephants in captivity is essential for laying a foundation for in-depth research on the intestinal microbial health of Asian elephants, rescue and captivity, and monitoring and protection, which is very important for the development of Asia elephants and the conservation of these endangered species. In this study, we aim to explore the gut microbiota of 29 healthy captive Asian elephants using feces samples from three different locations in Yunnan Province, China, using full-length 16S rRNA gene sequencing. We also explored several beneficial and opportunistic bacterial pathogens in the guts of Asian elephants. The retrieved microbial profiles from healthy captive elephants could reflect baseline data for wild and captive elephants. Revealing the microbiota could be invaluable information for elephant welfare assessment, leading to a guideline for feed preparation for improving their health and disease prevention.

## Materials and methods

### Study site and sample collection

A total of 29 fresh feces samples were collected from different captive Asian elephants in three main rescue centers located in Yuantongshan (102°71′N, 25°05′E) in Kunming (10 samples), Menghai (100°11′N, 22°04′E) in Menghai County (7 samples), and Yexianggu (100°3,411′N, 22°20′E) in Jinghong (12 samples) of Yunnan Province, China, from September 2022 to April 2023 (Table 1).

**Table 1:** Number of OTUs obtained from the three groups of Asian elephants.

Sample ID	No. of OTUs	No. of seqs
Yuantongshan	424	3,234
Menghai	479	3,653
Yexianggu	608	4,041

Asian elephants from Yuantongshan reside exclusively in enclosed enclosures, are occasionally exhibited to the public, and are allowed to be fed three times daily. The Menghai elephants are enclosed in a natural outdoor environment with ample space for movement and supplemented with plant feeding. The Yexianggu elephants are kept enclosed at night and, during the day, are led by caretakers to engage in outdoor activities. None of the Asian elephants at the three sites took antibiotics for 3 months. All 29 feces samples collected in separate 50-mL sterile centrifuge tubes before sunrise were stored in dry ice before being transported to the laboratory for DNA extraction.

### DNA extraction and full-length 16S rRNA gene amplification

All the collected fecal samples from each Asian elephant were subjected to microbial DNA extraction using the QIAamp DNA mini kit (Qiagen, United States), following the manufacturer’s protocol. Approximately 2 g of feces collected from the animals’ rectums were mixed with 5 mL of sterile cold normal saline. Centrifugation was performed at 13,000 rpm for 2 min, and the bacteria-containing pellets were collected. The collected pellets were used to extract microbial genomic DNA. The quality and quantity of the extracted DNA were assessed on a Nanodrop (ND-1000) spectrophotometer (Thermo Fisher Scientific, United States) and 1% agarose gel.

The full-length 16S rRNA gene was amplified using gene-specific PCR primers (27F: AGRGTTYGATYMTGGCTCAG; 1492R: RGYTACCTTGTTACGACTT) with a barcode. PCR amplification was performed by performing 35 cycles using the PCR Master Mix (Life Science, Shanghai, China). Initial denaturation was at 95°C for 2 min, followed by denaturation at 98°C for 10 s, annealing at 55°C for 30 s, extension at 72°C for 1 min and 30 s, and a final extension at 72°C for 10 min. The length and concentration of the PCR products were detected on a 1% agarose gel. PCR products were mixed in equidensity ratios according to the GeneToolsAnalysis software (v.4.03). Then, the mixture of PCR products was purified with the HiPure Gel Pure DNA Mini Kit.

### High-throughput sequencing

Following the amplification and purification of the 16S rRNA gene with Agencourt AMPure XP beads (Beckman Coulter, United States), the PCR amplified was quantified using the Qubit dsDNA assay kit and Qubit (v.3) fluorometer (Thermo Fisher Scientific, USA). Following quantification, the 16S rRNA amplicons were combined in equal quantities for library construction with the Prep Kit 2.0 (Biosciences, United States), as per the manufacturer’s guidelines. The constructed 16S rRNA gene libraries from each pooled and barcoded sample were sequenced on a PacBio Sequel II 8 M platform (Bioscience, United States), following company protocol.

### Data processing and bioinformatics analysis

PacBio sequenced data were processed using the SMRT tool (v.6.0), including data splitting and error correction. The clean data obtained from each sample were clustered into operational taxonomic units (OTUs) based on a 97% similarity threshold with the UPARSE Usearch software (v.10). During the clustering, UPARSE could simultaneously remove the chimera sequence and singleton OTU. The annotation of taxonomic information for each representative sequence was obtained by mapping with the Silva database (v.132). The R package was used to draw the histograms (heatmaps, ternary phase diagrams, etc.) based on the relative abundance of microbes at different taxonomic levels. To study the phylogenetic relationship of OTUs, the KRONA online tool was used to visualize the results of individual sample annotations. We evaluated the alpha diversity of the microbial communities using the Chao1, Shannon, and Simpson indices using QIIME (v.1.9.1). Additionally, we performed Kruskal–Wallis tests to examine differences in microbial diversity between groups. We used PCoA with Bray–Curtis and Euclidean dissimilarity estimates and tested the significance by analysis of similarities (ANOSIM). Linear discriminant analysis effect size (LEfSe) analysis (with an LDA score equal to or greater than 2; *p* < 0.05) revealed significantly abundant taxa among the groups.

## Results

### Full-length 16S rRNA gene sequencing data

After assembly and quality filtering, PacBio sequencing produced 365,655 clean reads with an average length of 1480.55 and a Q30 greater than 97.4% from all 29 fecal samples for downstream analyses (Supplementary Table S1). Clean tags from each sample were aggregated and filtered to generate OTUs at different taxonomic levels. Clustering at 97% identified 21,147 OTUs in all the samples. (Supplementary Table S2).

### Microbial community compositions in the fecal samples of *Elephas maximus*

**Figure 1:**
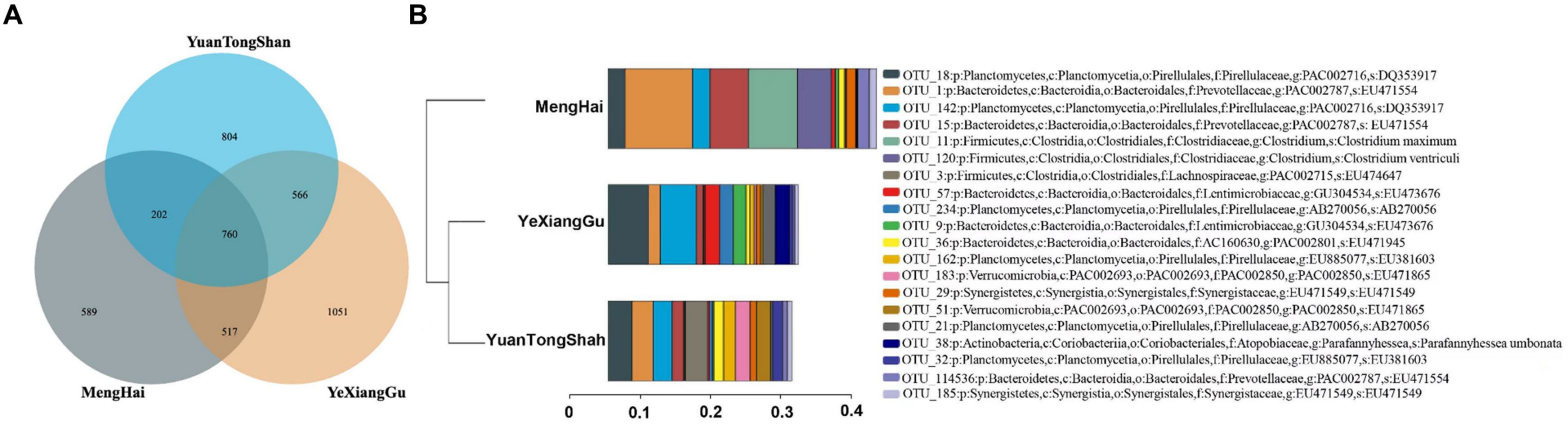
Microbial community compositions in the gut of Asian elephants from Yuantongshan, Menghai, and Yexianggu regions. **(A)** The Venn diagram shows that 21,147 OTUs were shared by Yuantongshan, Menghai, and Yexianggu data. **(B)** The details about the top 15 OTUs in the Yuantongshan, Menghai, and Yexianggu groups are shown.

To examine the microbial taxonomic differences in the Yuantongshan, Menghai, and Yexianggu Asian elephants, a total of 29 samples that passed the sequence quality filtering process generated 424 OTUs from the Yuantongshan, 479 from Menghai, and 609 from Yexianggu groups (Table 1). The Venn diagrams show 826 OTUs unique to the Yuantongshan, 572 to the Menghai, and 1,043 to the Yexianggu groups, whereas 778 OTUs were shared among the three groups (Figure 1A). The details about the top 15 OTUs among the Yuantongshan, Menghai, and Yexianggu groups are shown in Figure 1B. Two important *Clostridium* species (*Clostridium maximum* and *Clostridium ventriculi*) were found among the shared groups (Supplementary Table S3).

### Comparing the relative abundance of microbial profiles from three different locations

**Figure 2:**
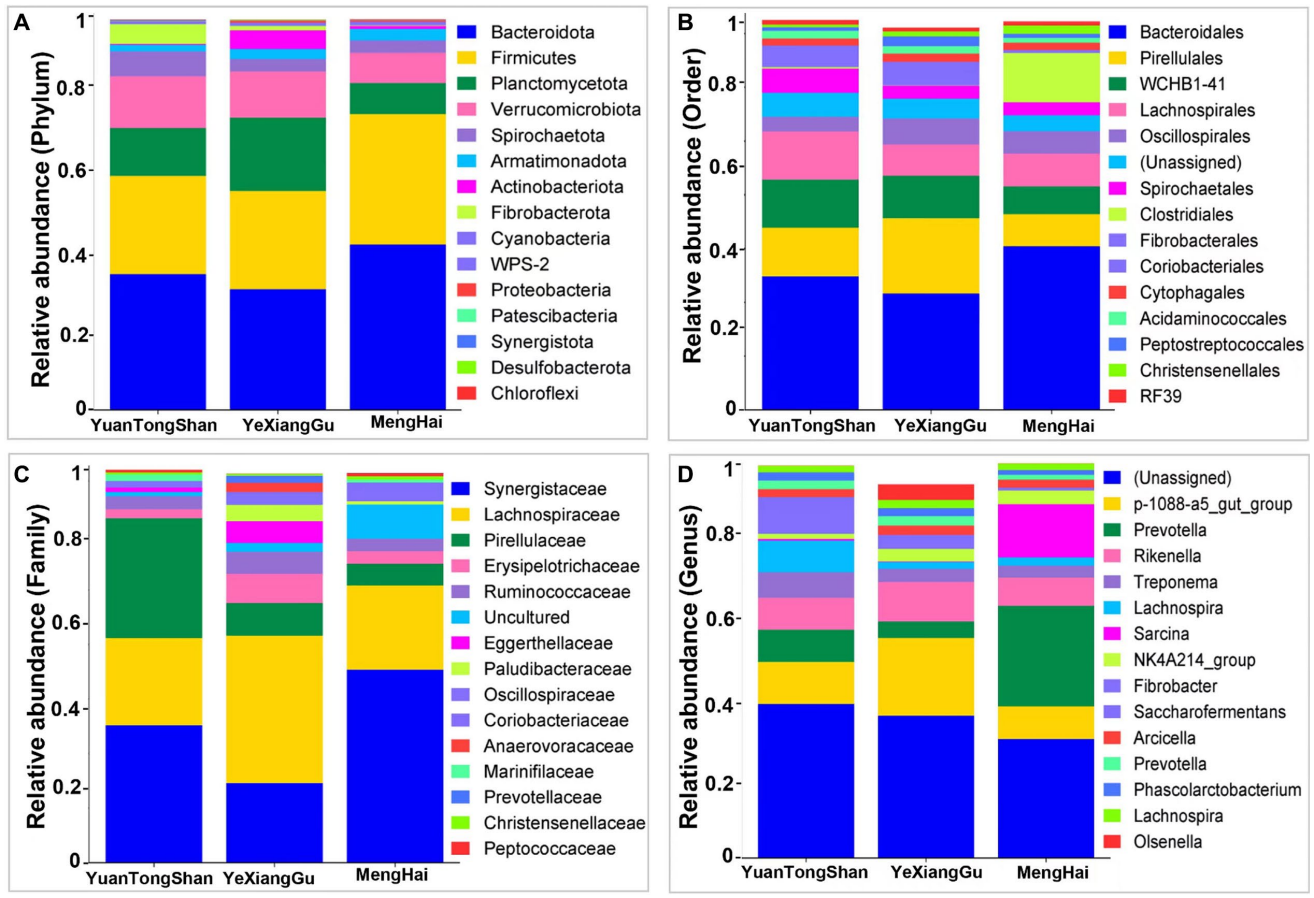
Relative microbial abundance at different taxonomic levels among the three groups. **(A)** Phylum, **(B)** order, **(C)** family, and **(D)** genus levels.

Distinct relative microbial abundance at different taxonomic levels was observed between the Yuantongshan, Menghai, and Yexianggu groups (Figure 2; Supplementary Table S4). According to the relative microbial abundance at the phylum level, Bacteroidetes, Firmicutes, Planctomycetota, and Verrucomicrobiota were higher in the elephant guts (Figure 2A). At the order level, Bacteroidales, Pirellulales, WCHB1-41, and Lachnospirales were higher among the three groups (Figure 2B). The family-level classification shows the highest abundance of Synergistaceae in the Menghai group, followed by Yuantongshan and Yexianggu. Lachnospiraceae was the richest in Yexianggu, whereas Pirellulaceae was the richest in the Yuantongshan group (Figure 2C). The highest relative abundance was unassigned among all three groups at the genus level. Genus p-1008-a5-gut-group was higher in Yexianggu than in the other two groups, whereas Prevotella was higher in the Menghai group (Figure 2D). Analysis at the species level revealed several beneficial and opportunistic bacteria in the guts of Asian elephants collected from three different locations. Bacterial species such as *Acinetobacter rathckeae*, *Bifidobacterium thermacidophilum*, *Clostridium maximum*, *Clostridium neonatale*, *Clostridium saccharoperbutylacetonicum*, *Enterococcus mundtii*, *Klebsiella huaxiensis*, *Lactobacillus agilis*, *Lactobacillus kitasatonis*, *Limosilactobacillus mucosae*, *Prolinoborus fasciculus*, and *Pseudoscardovia suis* were found in the Menghai group. *Faecalibacterium prausnitzii* and *Leuconostoc mesenteroides* were found in Yuantongshan. *Bacillus pumilus*, *Clostridium herbivorans*, *Collinsella aerofaciens*, *Corynebacterium nasicanis*, *Corynebacterium pollutisoli*, *Enterocloster alcoholdehydrogenati, Faecalibacterium prausnitzii*, *Holdemanella porci*, *Howardella ureilytica*, *Parafannyhessea umbonate*, *Streptococcus equinus*, *Streptococcus lutetiensis*, *Terrisporobacter mayombei*, *Tractidigestivibacter scatoligenes*, and *Weissella confusa* were found in the Yexianggu group. *Treponema* sp., *Clostridium methylpentosum*, was found in the Menghai group. *Clostridium chromiireducens*, *Clostridium butyricum*, *Clostridium phytofermentans, Incertae sedis*, *Lactobacillus fermentum*, *Lactobacillus sali*var*ius*, *Marseille*-P2968, and *Pseudobutyrivibrio ruminis* were shared among the three groups. The details about unique and shared bacterial species are shown in Supplementary Table S5.

### Microbial diversity

**Figure 3:**
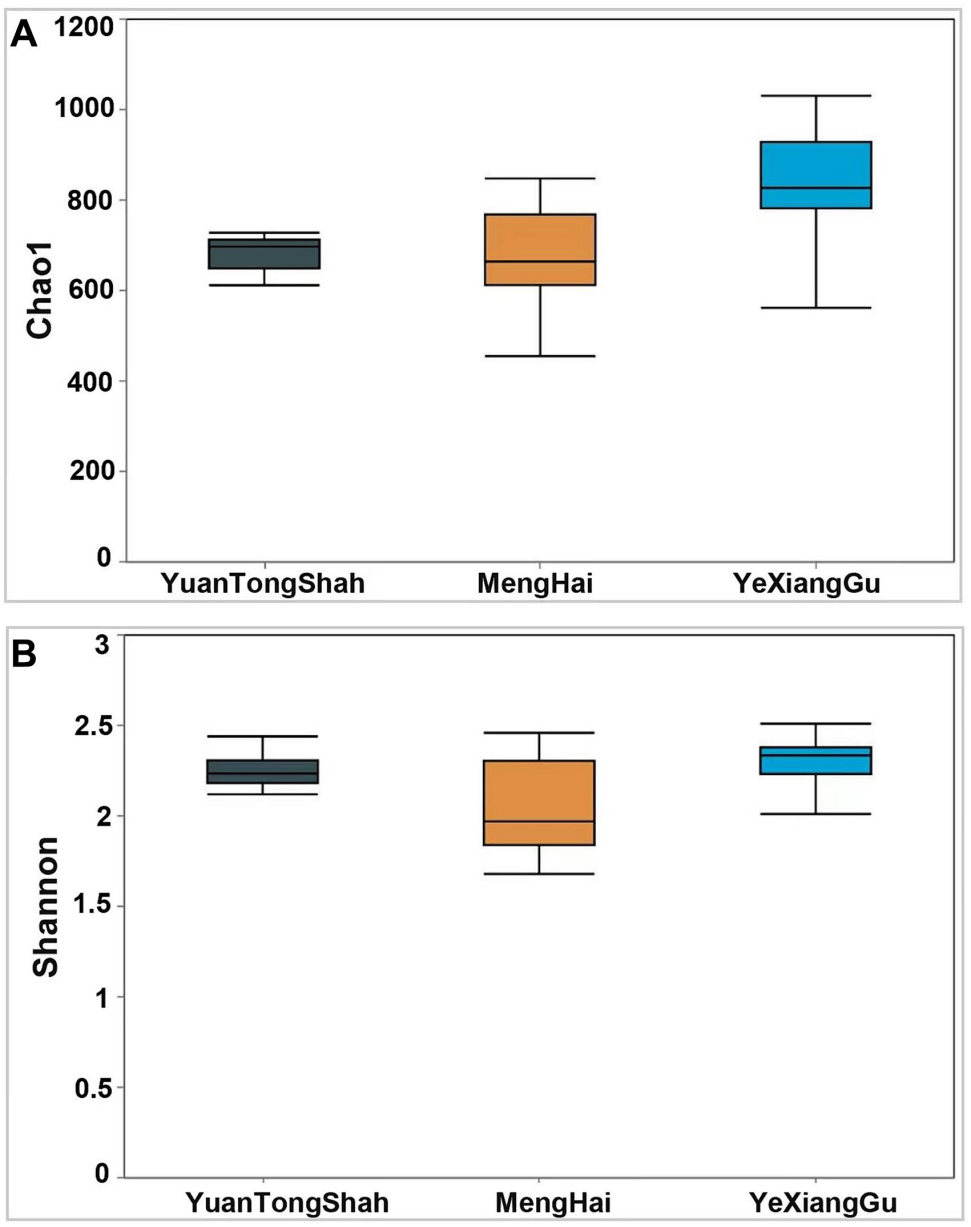
Boxplots show α-diversity among the three groups. The index of **(A)** Chao 1 and **(B)** Shannon. The Shannon and Simpson diversity index is a standard diversity measure that reflects the sample’s richness and evenness. The Chao 1 index is commonly used in ecology to estimate the total number of species. Larger Chao 1 values represent the total number of species. *p* < 0.05.

Microbial diversity analysis revealed distinctive microbial profiles among the fecal samples collected from different locations. Alpha diversity quantifies microbial differences between groups. The Chao1 (*p* = 0.005) and Shannon diversity indexes showed significant differences between the groups (Figures 3A,B). The Chao1 and Shannon diversity indices showed that the Yexianggu microbial communities tended to have higher alpha microbial diversity than the Yuantongshan and Menghai groups.

### Microbial variation

**Figure 4:**
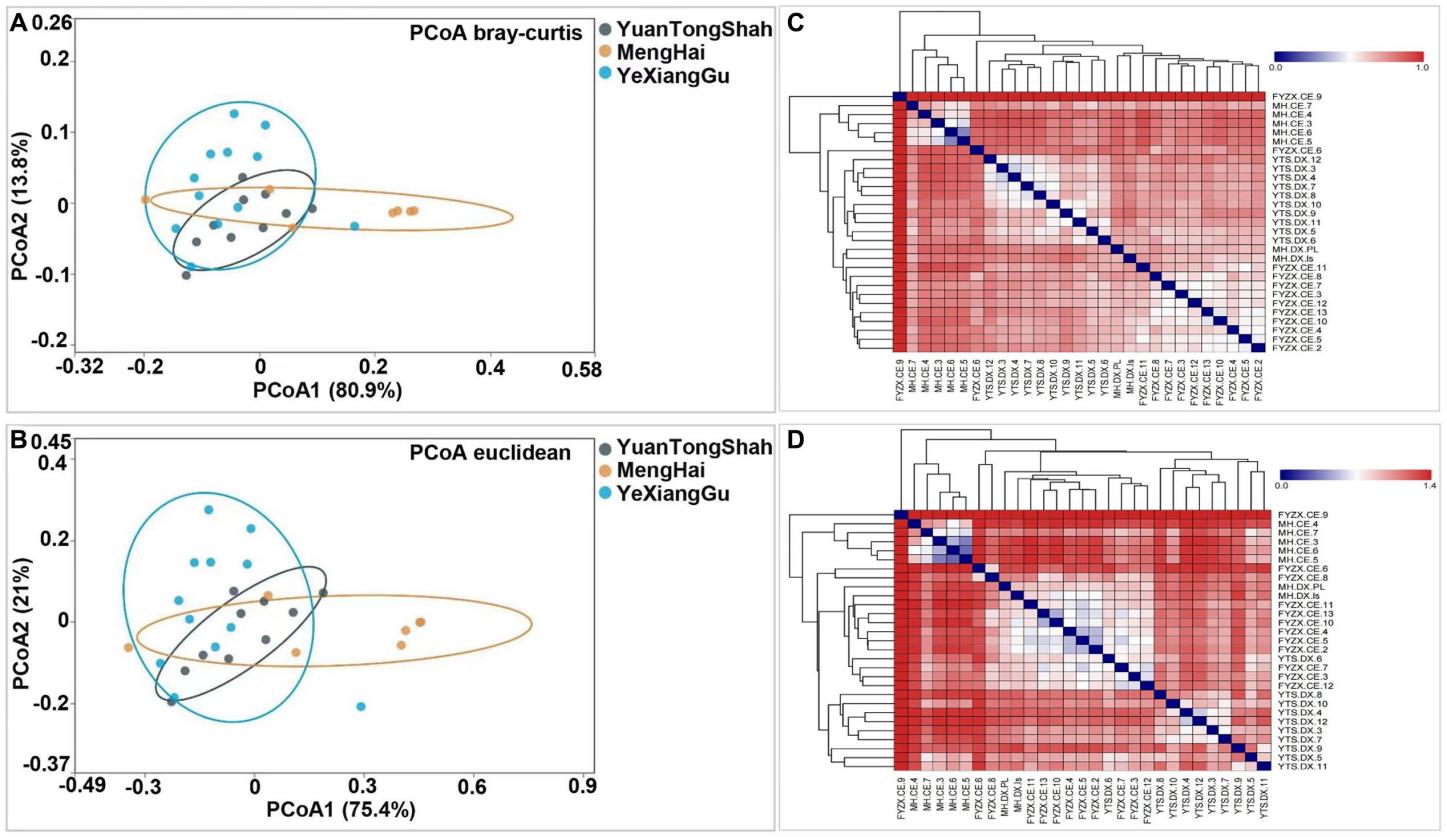
Bray–Curtis and Euclidean distance metrics revealed microbial similarities and differences among the Asian elephants of the Yuantongshan, Menghai, and Yexianggu groups. **(A)** Using the Bray–Curtis distance, PCoA plots visualize the percentage variations of PCoA1 (80.9%) and PCoA2 (13.8%) among different samples. **(B)** Two PCoA coordinates based on Euclidean distances revealed percentage variations of PCoA1 (75.4%) and PCoA2 (21.0%) among the samples. **(C)** Bray–Curtis distances. The most similarity and lowest difference in the bacterial community profile was 0.415 (shown with yellow triangles), observed between YTS.DX.4 and YTS.DX.3 (Yuantongshan samples). **(D)** The lowest difference was 0.686 between YTS.DX.3 and YTS.DX.7 (Yuantongshan samples).

A slight variation was observed between the samples from different regions. Testing beta diversity differences between groups of elephants based on Bray–Curtis and Euclidean distance metrics revealed significant differences in microbial variation between elephants from Yuantongshan, Menghai, and Yexianggu (*R*^2^ = 0.308, *p* = 0.002 and *R*^2^ = 0.31, *p* = 0.003). Two PCoA plots visualized the percentage variations of PCoA1 (80.9%) and PCoA2 (13.8%) among different groups using the Bray–Curtis distance metric (Figure 4A). In addition, two PCoA coordinates based on Euclidean distance revealed percentage variations of PCoA1 (75.4%) and PCoA2 (21%) among the three groups (Figure 4B), indicating that the visualized percentage variation may be attributed to differences in the feeding method conditions. Heatmaps based on the Bray–Curtis and Euclidean distance metrics further revealed microbial diversity and similarity among the Yuantongshan, Menghai, and Yexianggu groups. According to Bray–Curtis distance, the most similarity and lowest difference in the bacterial community profiles were 0.4152, observed between YTS.DX.4 and YTS.DX.3 (Yuantongshan) (Figure 4C), followed by 0.451 between YTS.DX.7 and YTS.DX.3 (Yuantongshan). The detailed values of the heatmaps based on the Bray–Curtis distance are shown in Supplementary Table S7. The lowest difference was 0.686, observed between YTS.DX.3 and YTS.DX.7 (Yuantongshan) (Figure 4D). The detailed values of the heatmaps based on the Euclidean distances are shown in Supplementary Table S8.

### Identification of potential microbial biomarkers

**Figure 5:**
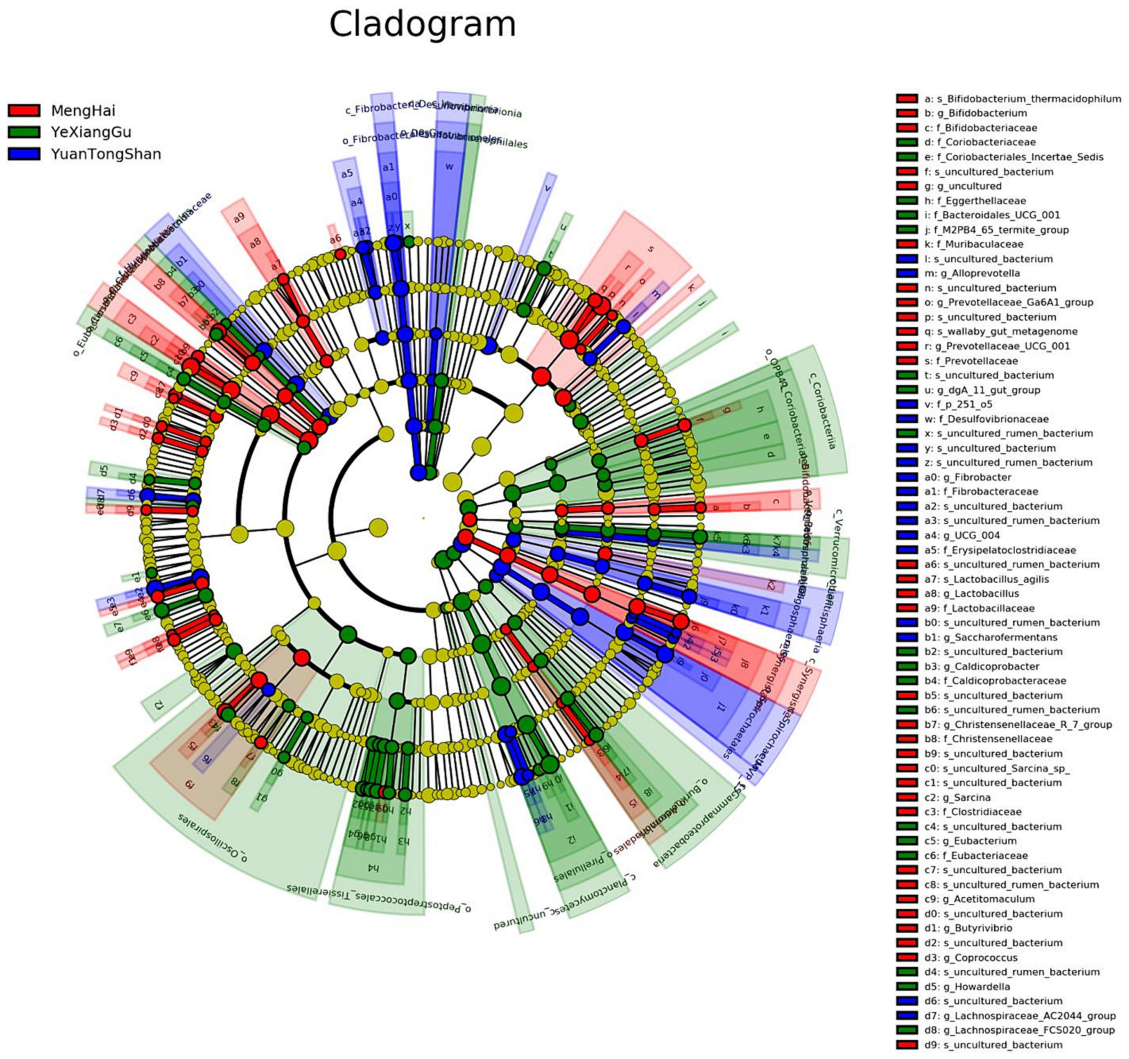
Overall, the cladogram shows 106 relatively abundant taxonomic clades (with an LDA score ≥ 2; *p* < 0.05) in Yuantongshan (blue), Menghai (red), and Yexianggu (green) groups. Of the total, 36 clades existed in Yuantongshan group, 27 in Menghai, and 43 in Yexianggu, consistent with the LEfSe findings.

Multivariate statistical analysis, LEfSe (*p* < 0.05; LDA score ≥ 2), analyzed several differentially abundant taxonomic biomarkers of the Menghai (red), Yexianggu (green), and Yuantongshan (blue) groups. The relative abundances of 43 taxonomic biomarkers were significantly higher in the Yexianggu group, followed by 36 in the Yuantongshan group and 27 in the Menghai group (Figure 5). The details about significantly abundant taxonomic biomarkers are shown in Supplementary Table S6.

### Random forest model construction and validation

**Figure 6:**
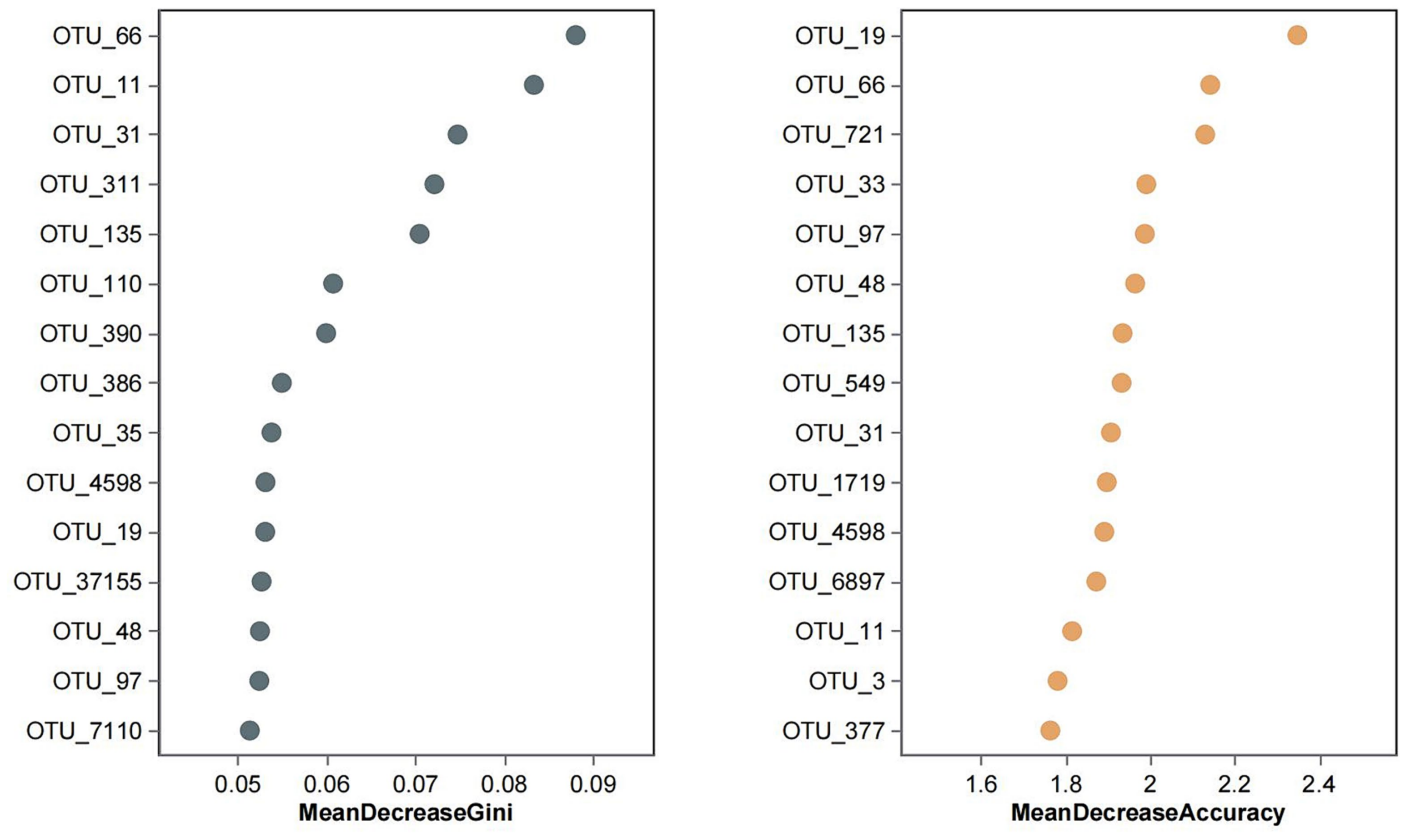
Random forest analysis identified the 15 most important OTUs with the highest discriminatory power among the three groups. Random forests of 10,000 trees were computed using the R package to generate the classifiers. The average mean decrease Gini and mean decrease accuracy values for each OTU were calculated, and the top 15 OTUs with the highest mean decrease Gini and mean decrease accuracy values were plotted. FYZX are wild rescue elephants and the offspring of wild rescue elephants, and the Asian elephants here are active in the enclosure at night, and 3 days a week are led by “elephant fathers” in the surrounding forests. MH wild Asian elephants are the problem group, they are confined to a natural habitat with natural vegetation and artificial nitrate ponds, the wild Asian elephants move freely in the temporary control area every day, and professional people come to a fixed place to drop food every day. Yuantongshan includes 10 wild Asian elephants, which is one of the largest wild Asian elephant populations in domestic urban zoos. Wild Asian elephants are released in a sandy place outdoors every day, and the rest of the time are kept in indoor captivity. In indoor enclosures, Asian elephants are separated, with one Asian elephant moving around in a small compartment and the freshest intact feces in each compartment collected the next day. The Asian elephants at all three sites received regular quarterly doses of Ivermectin tablets, and we collected stool samples close to their next dose to ensure the authenticity of their gut flora. Some species of *Clostridium* (e.g.*, Clostridium difficile*) can cause severe diarrhea and enteritis in the intestines of animals and may increase the risk of disease if co-existing with other pathogenic *Clostridium* species.

A random forest classifier was trained on the OTU abundance of the Yuantongshan, Menghai, and Yexianggu groups for discrimination analysis using the RandomForest package in the R program. To determine the optimal number of OTUs for model testing, 15 repeats of 10-fold cross-validation were performed. The importance of the random forest variable was calculated by calculating the mean decrease in Gini and accuracy (Figure 6). A random forest model was used to distinguish the Yuantongshan, Menghai, and Yexianggu groups. Gini and accuracy decrease with random forests. According to cross-validation, the three species are represented by black and brown circles.

## Discussion

According to this study, captive mode influences the gut microbiota environment of Asian elephants. Due to their habitat and diet differences, Asian and African elephants have evolved into two distinct species. These two factors may contribute to the difference in the gut microbial community during evolution (Terborgh et al., 2018). In this study, we analyzed the composition and abundance of the gut microbiota among the captive Asia elephants using full-length 16S rRNA gene sequencing. We found altered gut microbiota in captive Asian elephants. According to our findings, captivity, including encompassing diet and training, significantly impacts the alpha-diversities of Asian elephants in Yunnan province, China, as in the previous study (Keady et al., 2021). Notably, regardless of animal species (such as horses, cattle, or elephants), β-diversity was almost similar (Kartzinel et al., 2019; Keady et al., 2021), indicating influential environmental and nutritional factors.

According to the relative microbial abundance, the gut microbiota of captive Asian elephants was dominated by the phyla Bacteroidetes, Firmicutes, Planctomycetota, and Verrucomicrobiota in this study. Four bacterial phyla are core members of the mammalian gut microbiota, including Bacteroidetes and Firmicutes, consistent with previous research findings (Nishida and Ochman, 2018). Polysaccharide utilization loci were abundant in Bacteroidetes, suggesting that bacteria of this phylum are mainly involved in cellulose degradation in the Asian elephant (Ilmberger et al., 2014). Firmicutes mainly comprise cellulose-degrading bacteria that produce energy for the hosts. Such bacteria are likely responsible for colonic fermentation in Asian elephants by producing specific enzymes (Wood et al., 2020). Bacteroidetes and Firmicutes often appear in the intestines of herbivores. They can help degrade simple sugars, proteins, and carbohydrates. Bacteroidetes mainly ferment carbohydrates, polysaccharides, proteins, and bile acids for steroid metabolism, promote fat accumulation, develop the host immune system, enhance host immunity, and are especially essential for balancing the gut microbiota (Pryde et al., 2002). Firmicutes can degrade sugars and fats containing foods in the intestines to generate energy and other nutrients for the host. Therefore, they improve the host’s food utilization efficiency. In addition, Firmicutes metabolize carbohydrates to produce short-chain fatty acids, which are closely related to host immune regulation, compared to dominant phyla (Clostridiales and Prevotellaceae) in other household mammals (Warnecke et al., 2007). Our investigation found that the staple foods of Asian elephants from three groups are mainly Napier grass, sugarcane, banana, etc. Furthermore, Asian elephants in the Menghai group browsed *Musa acuminata* and shrubs. So we can find that the Bacteroidetes and Firmicutes abundance in the Menghai group elephants was higher than that in the other two groups, which may be due to the consumption of more carbohydrates similar to wild and captive golden snub-nosed monkeys (Wang et al., 2023). Captive farming approaches in Asian elephants reduce the abundance of Fibrobacteria and Verrucomicrobiota, Lachnospiraceae, and Ruminococcaceae (Zhang et al., 2023b).

Their lower abundance in captive elephants may be linked to a lower diversity in diet items compared to the wild (Thorel et al., 2023) and seasonal changes in food availability, which are dramatically reduced in the yearlong homogeneous pellet-based diet of captive-bred elephants (Wood et al., 2020). Our results also suggest that different captive modes might be the leading cause affecting the gut microbiota of Asian elephants under other circumstances. These diverse care models provide insights into the varied lifestyles of Asian elephants in captivity. The Yuantongshan elephants, confined to enclosed spaces, experience a more controlled environment with scheduled feeding and occasional public exposure. In contrast, the Menghai group enjoys a semi-wild experience with relative freedom to roam in a natural setting and access supplementary vegetation, reflecting a more naturalistic approach to captive care. The Yexianggu group’s elephants have a blend of enclosed and outdoor activities, allowing them to experience dynamic environments.

Daily life activity and captivity impact gut microbial diversity and composition (Wood et al., 2020). In our study, Synergistaceae and Lachnospiraceae were the most abundant in Asian elephant samples from Menghai and Yexianggu, whereas Pirellulaceae was the second largest family among the Yuantongshan group. Synergistaceae encode multiple pathways that may be associated with the metabolism of diet-generated compounds (Shiffman et al., 2017), and these are predicted to be critical factors in dietary detoxification in herbivores. In a study, Synergistaceae were significantly enriched in the goat milk-plant mixed-feed diet group, consistent with the significant enrichment of the biosynthesis of other secondary metabolites (Zhang et al., 2023a). This was likely due to this group’s excess secondary metabolism occurring during food digestion. In addition, Lachnospiraceae were more abundant in young Asian elephants in the milk-plant mixed feeding group compared to the goat milk-plant mixed-feed diet group and are closely associated with host mucosal integrity, bile acid metabolism, and polysaccharide catabolism (Lin et al., 2018). In contrast, the low Lachnospiraceae abundance in the goat milk-plant mixed-feed diet group (Zhang et al., 2023a) implies that goat milk may not be the best feeding choice for baby elephants. The significance enriched the Pirellulaceae abundance in the feces of the Yuantongshan group, which showed that a distinct microbial habitat supports a unique microbiota community structure, which may impact microbial communities’ ecological functions in different locations.

Interestingly, our findings revealed that despite differences in their habitats, captive Asian elephants shared some core genera among Yuantongshan, Yexianggu, and Menghai groups. The shared core microbiota, such as unassigned, p-1088-a5-gut_group, Prevotella, and Rikenella, among samples from different environments, indicates remarkable consistency in specific microbial genera. This finding suggests that while elephants in captivity settings experience contrasting diets and living conditions, there are underlying factors that drive diverse bacterial genera common to other herbivores, like in the feces of white rhinoceros (Bian et al., 2013), the large intestine of horses (Dougal et al., 2013), domesticated herbivorous ruminants, hindgut fermenters, and monogastric animals (O’Donnell et al., 2017). Species-level analysis revealed several beneficial and opportunistic bacteria in the guts of Asian elephants. These bacteria include *Acinetobacter rathckeae*, *Bifidobacterium thermacidophilum*, *Clostridium maximum*, *Enterococcus mundtii*, *Klebsiella huaxiensis*, *Lactobacillus agilis*, *Lactobacillus kitasatonis*, and *Pseudoscardovia suis* in the Menghai group. *Faecalibacterium prausnitzii* and *Leuconostoc mesenteroides* in Yuantongshan. *Bacillus pumilus*, *Clostridium herbivorans*, *Collinsella aerofaciens*, *Corynebacterium nasicanis*, *Corynebacterium pollutisoli*, *Enterocloster alcoholdehydrogenati, Faecalibacterium prausnitzii*, *Holdemanella porci*, *Howardella ureilytica*, *Parafannyhessea umbonate*, and *Streptococcus equinus* were in the Yexianggu group. *Treponema* sp. and *Clostridium methylpentosum* were found in the Menghai group. This implies that the evolutionary heritage of hindgut fermenters contributes to the establishment of core bacterial genera, emphasizing their important functions in herbivorous digestion.

The current study has several limitations. The first is the small sample size and divergent sampling methods among Asian elephants (sterile collection of 30–40 g of fresh feces) (Budd et al., 2020). Another limitation is the V1–V9 region of the 16S rRNA gene with short-read sequencing platforms, which cannot achieve the taxonomic resolution afforded by sequencing. We implemented a consistent bioinformatics pipeline for data analysis to ensure the rigor and validity of comparative microbiota from different regional groups. For example, we minimized noise with DADA2 software and taxonomic assignments using the SILVA database and statistical analysis, which made fast and accurate sample inferences from amplicon data with single-nucleotide resolution.

## Conclusion

In conclusion, we explored microbial diversity and the impact of different environments on the gut microbiota compositions of Asian elephants. Understanding microbial variations is crucial for refining captive care practices and ensuring the health and happiness of these endangered giant mammals. Our findings provide critical insights for improving the management of these animals in captivity. Still, they contribute to a relatively new field of study: the intricate ecosystems formed by host-associated microbiota. Hence, it is clear that Asian elephants, regardless of habitat, share a fundamental gut microbiota associated with hindgut fermenters.

## Data Availability

The data supporting the findings of this study are available in the NCBI repository, under the accession number PRJNA1151974. The data can be accessed via the following link: https://www.ncbi.nlm.nih.gov/bioproject/PRJNA1151974 ID1151974 - BioProject - NCBI (nih.gov).

## References

[ref1] Bian G. Ma L. Su Y. Zhu W. (2013). The microbial community in the feces of the white rhinoceros (*Ceratotherium simum*) as determined by barcoded pyrosequencing analysis. PLoS One 8:e70103. doi: 10.1371/journal.pone.0070103 23922920 PMC3724812

[ref2] Budd K. Gunn J. C. Finch T. Klymus K. Sitati N. Eggert L. S. (2020). Effects of diet, habitat, and phylogeny on the fecal microbiome of wild African savanna (*Loxodonta africana*) and forest elephants (*L. cyclotis*). Ecol. Evol. 10, 5637–5650. doi: 10.1002/ece3.6305 32607180 PMC7319146

[ref3] Chen Y. Sun Y. Hua M. Shi K. Dudgeon D. (2023). Using genetic tools to inform conservation of fragmented populations of Asian elephants (*Elephas maximus*) across their range in China. Integr Zool 18, 453–468. doi: 10.1111/1749-4877.12680 36052971

[ref4] Davidar P. Sharma R. de Silva S. Campos-Arceiz A. Goossens B. Puyravaud J. P.. (2023). Connect elephant habitats in Asia. Science 379:765. doi: 10.1126/science.adg7470, PMID: 36821683

[ref5] Dougal K. de la Fuente G. Harris P. A. Girdwood S. E. Pinloche E. Newbold C. J. (2013). Identification of a core bacterial community within the large intestine of the horse. PLoS One 8:e77660. doi: 10.1371/journal.pone.0077660 24204908 PMC3812009

[ref6] Fraser D. J. (2008). How well can captive breeding programs conserve biodiversity? A review of salmonids. Evol. Appl. 1, 535–586. doi: 10.1111/j.1752-4571.2008.00036.x, PMID: 25567798 PMC3352391

[ref7] Ilmberger N. Güllert S. Dannenberg J. Rabausch U. Torres J. Wemheuer B.. (2014). A comparative metagenome survey of the fecal microbiota of a breast-and a plant-fed Asian elephant reveals an unexpectedly high diversity of glycoside hydrolase family enzymes. PLoS One 9:e106707. doi: 10.1371/journal.pone.0106707, PMID: 25208077 PMC4160196

[ref8] Kandel S. Sripiboon S. Jenjaroenpun P. Ussery D. W. Nookaew I. Robeson M. S.. (2020). 16S rRNA gene amplicon profiling of baby and adult captive elephants in Thailand. Microbiol. Resour. Announc. 9, 14–20. doi: 10.1128/mra.00248-20, PMID: 32527771 PMC7291096

[ref9] Kartzinel T. R. Hsing J. C. Musili P. M. Brown B. R. P. Pringle R. M. (2019). Covariation of diet and gut microbiome in African megafauna. Proc. Natl. Acad. Sci. USA 116, 23588–23593. doi: 10.1073/pnas.1905666116 31685619 PMC6876249

[ref10] Keady M. M. Prado N. Lim H. C. Brown J. Paris S. Muletz-Wolz C. R. (2021). Clinical health issues, reproductive hormones, and metabolic hormones associated with gut microbiome structure in African and Asian elephants. Anim. Microbiome 3:85. doi: 10.1186/s42523-021-00146-9 34930501 PMC8686393

[ref11] Klinsawat W. Uthaipaisanwong P. Jenjaroenpun P. Sripiboon S. Wongsurawat T. Kusonmano K. (2023). Microbiome variations among age classes and diets of captive Asian elephants (*Elephas maximus*) in Thailand using full-length 16S rRNA nanopore sequencing. Sci. Rep. 13:17685. doi: 10.1038/s41598-023-44981-z, PMID: 37848699 PMC10582034

[ref12] Li X. Chen J. Zhang C. Zhang S. Shen Q. Wang B.. (2023). Fecal metagenomics study reveals that a low-Fiber diet drives the migration of wild Asian elephants in Xishuangbanna, China. Animals 13:3193. doi: 10.3390/ani13203193, PMID: 37893918 PMC10603651

[ref13] Li G. Jiang Y. Li Q. An D. Bao M. Lang L.. (2022). Comparative and functional analyses of fecal microbiome in Asian elephants. Antonie Van Leeuwenhoek 115, 1187–1202. doi: 10.1007/s10482-022-01757-1, PMID: 35902439

[ref14] Lin Z. Ye W. Zu X. Xie H. Li H. Li Y.. (2018). Integrative metabolic and microbial profiling on patients with spleen-yang-deficiency syndrome. Sci. Rep. 8:6619. doi: 10.1038/s41598-018-24130-7, PMID: 29700349 PMC5920061

[ref15] Morfeld K. A. Meehan C. L. Hogan J. N. Brown J. L. (2016). Assessment of body condition in African (*Loxodonta africana*) and Asian (*Elephas maximus*) elephants in north American zoos and management practices associated with high body condition scores. PLoS One 11:e0155146. doi: 10.1371/journal.pone.0155146 27415629 PMC4944958

[ref16] Moustafa M. A. M. Chel H. M. Thu M. J. Bawm S. Htun L. L. Win M. M.. (2021). Anthropogenic interferences lead to gut microbiome dysbiosis in Asian elephants and may alter adaptation processes to surrounding environments. Sci. Rep. 11:741. doi: 10.1038/s41598-020-80537-1, PMID: 33436882 PMC7803949

[ref17] Nishida A. H. Ochman H. (2018). Rates of gut microbiome divergence in mammals. Mol. Ecol. 27, 1884–1897. doi: 10.1111/mec.14473, PMID: 29290090 PMC5935551

[ref18] O’Donnell M. M. Harris H. M. Ross R. P. O'Toole P. W. (2017). Core fecal microbiota of domesticated herbivorous ruminant, hindgut fermenters, and monogastric animals. Microbiology 6:e00509. doi: 10.1002/mbo3.509, PMID: 28834331 PMC5635170

[ref19] Pryde S. E. Duncan S. H. Hold G. L. Stewart C. S. Flint H. J. (2002). The microbiology of butyrate formation in the human colon. FEMS Microbiol. Lett. 217, 133–139. doi: 10.1111/j.1574-6968.2002.tb11467.x 12480096

[ref20] Shi H. T. Wang J. Chen H. Q. Parham J. F. (2021). China's wildlife protection: add annual reviews and oversight. Nature 592:685. doi: 10.1038/d41586-021-01111-x 33907328

[ref21] Shiffman M. E. Soo R. M. Dennis P. G. Morrison M. Tyson G. W. Hugenholtz P. (2017). Gene and genome-centric analyses of koala and wombat fecal microbiomes point to metabolic specialization for eucalyptus digestion. PeerJ 5:e4075. doi: 10.7717/peerj.4075, PMID: 29177117 PMC5697889

[ref22] Struckmann Poulsen J. de Jonge N. Vieira Macêdo W. Rask Dalby F. Feilberg A. Lund Nielsen J. (2022). Characterisation of cellulose-degrading organisms in an anaerobic digester. Bioresour. Technol. 351:126933. doi: 10.1016/j.biortech.2022.126933 35247567

[ref23] Terborgh J. Davenport L. C. Ong L. Campos-Arceiz A. (2018). Foraging impacts of Asian megafauna on tropical rain forest structure and biodiversity. Biotropica 50, 84–89. doi: 10.1111/btp.12488

[ref24] Thorel M. Obregon D. Mulot B. Maitre A. Mateos-Hernandez L. Moalic P. Y.. (2023). Conserved core microbiota in managed and free-ranging *Loxodonta africana* elephants. Front. Microbiol. 14:1247719. doi: 10.3389/fmicb.2023.1247719, PMID: 37860133 PMC10582353

[ref25] Wang Y. Yang X. Zhang M. Pan H. (2023). Comparative analysis of gut microbiota between wild and captive Golden snub-nosed monkeys. Animals 13:1625. doi: 10.3390/ani13101625, PMID: 37238055 PMC10215246

[ref26] Warnecke F. Luginbühl P. Ivanova N. Ghassemian M. Richardson T. H. Stege J. T.. (2007). Metagenomic and functional analysis of hindgut microbiota of a wood-feeding higher termite. Nature 450, 560–565. doi: 10.1038/nature06269, PMID: 18033299

[ref27] Williams C. Tiwari S. K. Goswami V. R. de Silva S. Kumar A. Baskaran N.. (2020). *Elephas maximus*. The IUCN red list of threatened species 2020: E. T7140A45818198. doi: 10.2305/IUCN.UK.2020-3.RLTS.T7140A45818198.en

[ref28] Wood J. Koutsos E. Kendall C. J. Minter L. J. Tollefson T. N. Heugten K. A. (2020). Analyses of African elephant (*Loxodonta africana*) diet with various browse and pellet inclusion levels. Zoo Biol. 39, 37–50. doi: 10.1002/zoo.21522 31710122

[ref29] Zhang C. Chen J. Wu Q. Xu B. Huang Z. (2023a). The gut microbiota of young Asian elephants with different Milk-containing diets. Animals 13:916. doi: 10.3390/ani13050916, PMID: 36899773 PMC10000238

[ref30] Zhang C. Lian Z. Xu B. Shen Q. Bao M. Huang Z.. (2023b). Gut microbiome variation along a lifestyle gradient reveals threats faced by Asian elephants. Genomics Proteomics Bioinformatics 21, 150–163. doi: 10.1016/j.gpb.2023.04.003, PMID: 37088195 PMC10372918

